# Edible Insects and Sustainable Development Goals

**DOI:** 10.3390/insects12060557

**Published:** 2021-06-15

**Authors:** Roberta Moruzzo, Simone Mancini, Alessandra Guidi

**Affiliations:** 1 Department of Veterinary Sciences, University of Pisa, Viale delle Piagge 2, 56124 Pisa, Italy; roberta.moruzzo@unipi.it (R.M.); alessandra.guidi@unipi.it (A.G.); 2 Interdepartmental Research Center “Nutraceuticals and Food for Health”, University of Pisa, Via del Borghetto 80, 56124 Pisa, Italy

**Keywords:** insect farming, SDGs, hunger, security, sustainability

## Abstract

**Simple Summary:**

The United Nations Sustainable Development Goals (SDGs), seventeen urgent topics of action by all country, aim to reach ambitious and hopefully targets, such as peace and prosperity for people and the planet, now and into the future. Edible insects were individuated as a potential response to one of the major challenges of our times: increasing food production while decreasing environmental impact. In this review, the “insect idea” was linked to the single SDGs in order to express its potentiality. Likewise, indirect linking between insect farming and several SDGs was reported.

**Abstract:**

The insect sector can become an important component of sustainable circular agriculture by closing nutrient and energy cycles, fostering food security, and minimising climate change and biodiversity loss, thereby contributing to SDGs. The high levels of the interaction of the insect sector with the SDGs is clearly illustrated inside the review, analysing all of the SDGs that can have direct and indirect effects on insects. Mapping the interactions between the SDGs goals and insect sector offers a starting point, from which it could be possible to define practical next steps for better insect policy.

## 1. Introduction

In the global sustainable development agenda, the United Nations’ (UN) “Transforming Our World: The 2030 Agenda for Sustainable Development” adopted 17 Sustainable Development Goals (SDGs) that are intended to “stimulate action over the next 15 years in areas of critical importance for humanity and the planet”. In policy circles, these SDGs are being increasingly referred to simply as “The Global Goals” [1]. They represent a global agreement across United Nation’s member states “used in national development plans, academic and foreign aid prioritization” [2]. As reported by Waage et al. [3], the 17 SDGs, with 169 targets and 232 specific indicators, can be represented in three concentric layers, which reflect their main intended outcomes: the wellbeing goals (i.e., 1, 3, 4, 5, 10, 16 SDGs), the infrastructure goals (i.e., 2, 6, 7, 8, 9, 11, 12 SDGs), and the natural environment goals (i.e., 13, 14, 15 SDGs). According to the FAO, food production will have to increase by 70% to be able to feed the world in 2050; growing population, increasing wealth, and urbanization, especially in recent industrialized countries, have changed consumption patterns and food preferences, leading to higher animal protein demands. This context places heavy pressure on already limited resources, aggravates the competition for land to produce food, feed, and fuel, and makes the challenge of environmental sustainability even more difficult. Conversely, while one-third of food is wasted [4], 8.9% of the world population are estimated to have been undernourished and 25.9% suffered from both moderate and severe levels of food insecurity in 2019. Insects reduce the above-mentioned societal challenges, create healthier and more sustainable food, and reduce animal feed production and consumption. Insects are rich in proteins (37–63%) and fats (20–40%), with well-balanced amino acid and fatty acid profiles, respectively, and they are good sources of minerals and vitamins [5]. When compared to conventional livestock, insect’ production has a lower environmental impact because of, amongst other things, the limited need for land and water and the reduction in greenhouse gas and carbon dioxide emissions [6,7]. As poikilotherm, insects have a high feed conversion rate, requiring much less feed to produce the same amount of animal proteins: 1 kg of live animal weight of crickets requires 1.7 kg of feed, as compared to 2.5 kg for chicken, 5 kg for pork, and 10 kg for beef [8,9]. In addition, insects have a higher percentage of edible mass, up to 80% when compared to around 55% of chicken, 70% of pork, and only 40% of cattle. Insects can also be cultured on locally available industrial and agricultural waste streams, recycling a loss into a valuable protein source. Moreover, insects can be gathered from nature or farmed with simple techniques and minimal facilities requiring minimal land or capital and have a quick growth rate [5]. 

**Figure 1:**
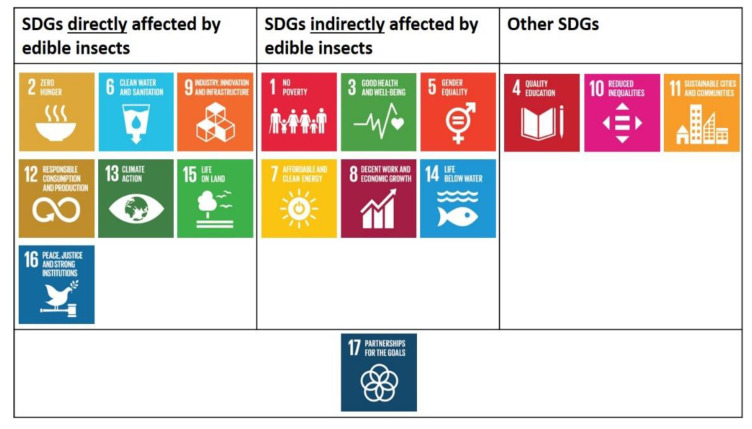
Interaction map between insects and sustainable development goals.

All of the above-mentioned insect features point out the high potentiality of this emerging sector. Insect farming will surely increase the overall agricultural production, both via large- and small-scale farms. An increased and widespread consciousness regarding the potential of insects will also contribute to political and marketing choices, contributing to increase livelihood, economic development, and social integration, especially in countries with a long tradition of entomophagy and insects rearing, such as Asia, South America, and Africa. A practical example of the insect potential is represented by the one called “The Thai example”. Indeed, even if the use of insect as food was historically present in the country, in the last decades it was only improved by moving from collecting insects into the wild to rearing them in close environment. It was proficiently driven by a strong market demand supported by academic research and innovation in private sectors (from processing to selling). A new production section was proficiently established that assured new incomes and employment to Thai people with more than 20,000 family farms rearing insects as food and feed [10]. Implementing innovative and sustainable food production strategies, such as insect farming, may contribute to several of the SDGs, which are interconnected [6,11,12,13,14]. Insect farming could directly or indirectly contribute to several SDGs, as proposed by different Authors [6,12,13]. Therefore, the aim of this review was to analyse all of the SDGs one-by-one and relate them to edible insects, referring to direct-indirect effects (Figure 1). Links between SDGs were also reported in order to improve the outcomes and contributions to reach the goals.

## 2. SDGs Directly Affected by Edible Insects

### 2.1. SDG 2. End Hunger, Achieve Food Security, and Improved Nutrition and Promote Sustainable Agriculture

The use of insects as a major feed component could reduce the dependence on expensive and imported feed, which is a bottleneck for market entry by small-scale livestock farmers. Additionally, by culturing insects on local waste streams, any leftovers can be used as bio fertilizer, which increases farmers’ independence from externally derived fertilizers [14]. Insects are highly appreciated as edible food in many parts of the world, except in most Western Countries. However, in recent years, western cultures have reconsidered entomophagy as part of a healthier and environmentally friendly diet. This has helped to push new global research, technological transfers, and international cooperation in the field [15,16]. In this context, there are chances for insects to become a healthy and sustainable food and feed alternative able to stimulate a proactive dialogue and operative actions between industrialized, developing, and least developed countries. In particular, insect gathering, rearing, and processing could act as important driving forces to ensure access to nutritious and sufficient food, to support economic development, fair market access, and social inclusion for disadvantaged categories. However, on a larger scale, it is important to work on the legal framework and the harmonization of a differentiated legal status of insects as food and feed across the world to facilitate the global use of this sustainable source and promote investments on both household and industrial production.

### 2.2. SDG 6. Ensure Availability and Sustainable Management of Water and Sanitation for All

Insects could contribute to improved water sanitation when they are directly reared on human–animals’ faeces. Several insect groups, mainly consisting of flies, already use faeces as the main nutritional resource for their larval or adult stages. Using faeces as a substrate of insects contributes to reducing pollution, which increases the sanitation of these materials. Moreover, South African studies highlighted the potential of black soldier fly larvae in faecal sludge management on treating urine diversion dehydrating toilets [17,18]. In this contest, some studies reported the capability of BSF to be reared on fresh human faeces [19,20,21,22]. Similarly, animal manure derived from zootechnical productions (dairy, chicken, pig) was tested with positive results [23,24,25,26,27,28]. Insects that were reared on these substrates could be used as feed or for the production of energy (e.g., biofuel), and their faeces could also be employed as fertiliser [24,27]. Clearly, hygiene and legislation issues could arise using these waste materials as rearing substrate, mostly for insects that are intended to be used as feed (food); therefore, national laws and hygiene practices must be taken into account [20,29]. Some research studies even reported a positive reduction of pathogens or at least the lack of bioaccumulation of dioxins, PCBs, and PAHs, and selected pesticides, pharmaceuticals, and mycotoxins in BSF larvae [20,30,31,32]. Contrarily bioaccumulation might be present in BSF larvae in relation to the cadmium, lead, mercury, zinc, and arsenic up take [33,34]. Notably, insects could also eventually be used for energy production in the case of dangerous bioaccumulations, contributing to the reduction of pollution. Not least, if compared to conventional agricultural-zootechnical productions, feed-food goods that are derived from insects could contribute to decreasing the quantity of water required in the farming systems. Indeed, the low amount of water requested by insects (in some cases, even not required as furnished with moisture present in the solid feeds) could reduce water scarcity without affecting feed–food productions [5].

### 2.3. SDG 9. Build Resilient Infrastructure, Promote Inclusive and Sustainable Industrialization and Foster Innovation

Edible insects can be obtained in three ways: (I) wild harvesting; (II) semi-domestication (habitat manipulation to increase production); and, (III) farming, which can range from the single small cage scale to a large factory [35]. When only considering edible insects’ human consumption (entomophagy), wild harvesting represents about 92%, while semi-domesticated insects only constitutes 6%, according to Yen. Therefore, only 2% of edible insects are currently farmed. However, insects may not be available all year round in the wild due to seasonal and geographical variations. Therefore, industrial scale insect production, helped by sustainable insect breeding, farming, and processing technologies, can ease the constraints of insect availability, and lower the sale price of edible insects. It is noteworthy that, so far, farming is the most efficient way to produce insects that are intended as feed and food [36], and it could create a novel economic sector with standardized techniques on industrial scale [5]. Furthermore, farming insects in small to medium enterprise levels could contribute to producing high nutritional food in time-efficient and low technology practice [37]. Indeed, this kind of farming allows fast investment and high financial returns, due to the limited investment costs (unit/protein produced), the relatively simple management (not require in-depth training), the rapid production cycle, and the high feed conversion efficiency (with a low environmental impact if reared on side stream substrates). Low-income communities may be positively influenced via increased employment and the development of local technology [11]. Furthermore, in countries where edible insects are traditionally harvested, this practice is a source of familiar income and a socially important role, as reported by van Huis & Oonincx [38].

### 2.4. SDG 12. Ensure Sustainable Consumption and Production Patterns

The goal of a sustainable consumption and production system is to transform energy and materials to maintain or even improve human wellbeing without negatively impacting environmental resources [39]. Food loss and waste are serious threats to the sustainability of our food systems. Roughly one-third of the global food production for human consumption (c.a. 1.3 billion tonnes per year) is lost or wasted, according to the Food and Agriculture Organisation of the United Nations (FAO) [40]. One of the SDG 12 target is to “halve the per capita global food waste at the retail and consumer levels and reduce food losses along production and supply chains, including post-harvest losses”. The concept of Circular Economy (CE) can offer tools to enhance and optimise the sustainability of a food system [41]. The importance of insects in CE has been pointed out by different authors highlighting that insect farming is an advantageous choice within a CE [42,43]. In particular, using the food waste for rearing insects provides an attractive key for closing the loop of food value chain [44]. Insect farming can be easily practiced through recycling food and organic waste without having remarkable implications on productivity and quality [42]. Surely, it is useful to adopt life-cycle assessments in order to analyse the direct and indirect effects of insects in CE perspective [7,38,45]. Life-cycle assessments can have direct and indirect consequences that may result in a trade-off between the effects on land use and climate change mitigation [46]. We have to balance and carefully assess all the factors, as correctly reported by Dicke [6]. If, on one hand, waste streams could be used in insect farming for feed-food purposes, lowering land use and losses, on the other hand, we may subtract these materials to other productions, such as bio-energy production. If this energy production is overcome by fossil fuels instead of solar energy, all the effects will be lost.

### 2.5. SDG 13. Take Urgent Action to Combat Climate Change and Its Impacts

Livestock contribute drastically to the global greenhouse gas (GHG) emissions, directly and indirectly. In the publication entitled “Livestock’s long shadow: environmental issues and options”, the FAO directly attribute about 9 percent of total carbon dioxide emissions (not considering respiration), 37 percent of methane (rumen fermentation), and 65 percent of nitrous oxide (including feed crops) to livestock [47]. This equates to an approximate total livestock contribution of about 18% to climate change in CO_2_ equivalent (including pasture degradation and land use change). Insects produce very low amounts of greenhouse gases and ammonia. Oonincx et al. [48] conducted a cost–benefit analysis of five edible insects rearing systems intended as greenhouse gases production (environmental cost) and food production (benefit). Four insect species (*Tenebrio molitor*, *Blaptica dubia*, *Acheta domesticus* and *Locusta migratoria*) reported less greenhouse gas emissions than pigs and ruminants (based on kg of mass gain). Similarly, the ammonia levels of insect farming were lower than conventional livestock. It is important to highlight that farming systems could also affect GHG emissions, both in regard to insects and conventional livestock. On the other hand, insects could become a potential resilient mini-livestock production in those countries that are currently facing climate changes. The low required amount of water, the capability to be reared on side streams, and the ability to grow proficiently in hot climates could also contribute to ensuring animal production in harsh climates.

### 2.6. SDG 15. Protect, Restore and Promote Sustainable Use of Terrestrial Ecosystems, Sustainably Manage Forests, Combat Desertification, and Halt and Reverse Land Degradation and Halt Biodiversity Loss

Insect framing could contribute to mitigating biodiversity reduction in two ways. Firstly, insect rearing uses a small amount of land, as most of them can be reared in a close environment using horizontal and vertical farming space [49]. Moreover, insect rearing does not need direct contact with the ground and soil, and it could be done in an anthropized environment, taking advantage of already exploited locations. Thus, if insects are intended as substitution of the conventional livestock, it will induce a decrease in land use and even in restoring ecosystems. Secondly, insects could be used as feed in several other livestock production. Feed supply chain is one of the most significant environmental burdens in animal farming; reducing plant production for feed, such as soybean and corn, could contribute to increased biodiversity and combatting desertification [47]. It is noteworthy that the potential use of side stream materials as substrate for insects will also increase the circular economy, reducing total land use. Finally, increased consciousness regarding insects’ features could contribute to making people more careful and respectful of wildlife animals and increase the protection of biodiversity.

### 2.7. SDG 16. Promote Peaceful and Inclusive Societies for Sustainable Development, Provide Access to Justice for All and Build Effective, Accountable and Inclusive Institutions at All Levels

The promotion of peaceful and inclusive societies has a strong and direct connection with the goals of food security and zero hunger. Conflicts can be the cause of food insecurity as well as a consequence when food prices increase during times of social instability and reduced food availability. Nutritional deficiencies, above all protein, emerge among disadvantaged segments of society, and smallholding farmers, on which global food production largely depends, suffer the most during times of conflict [6]. The gathering, rearing, and processing of insects offer an important tool for supporting the peace process, fighting food insecurity due to their nutritional composition, accessibility, easy and cheap rearing techniques, and the possibility to be reared on organic residual streams by-producing biofertilizer. For these reasons, the development of the production of these sustainable proteins can improve the living condition and social stability of smallholder farmers as well as vulnerable people, such as women and landless people in urban and rural areas of developing countries, which would make a valuable contribution to their economic independency and social integration [5,12,14]. Therefore, insects may yield a local circular economy and contribute to promoting and maintaining peaceful and inclusive societies, reducing the risks of riots and violence. However, food security is a shared responsibility, and it is important that different stakeholders are involved in the process. In this case, governments, as well as other institutions, among them universities and private companies, should be involved in national and international capacity building and networking projects, and be effective in local farmers information and training in order to develop the insect-derived food and feed sector, especially in developing countries. 

## 3. Links between SDGs (Directly-Indirectly Affected by Edible Insects)

**Figure 2:**
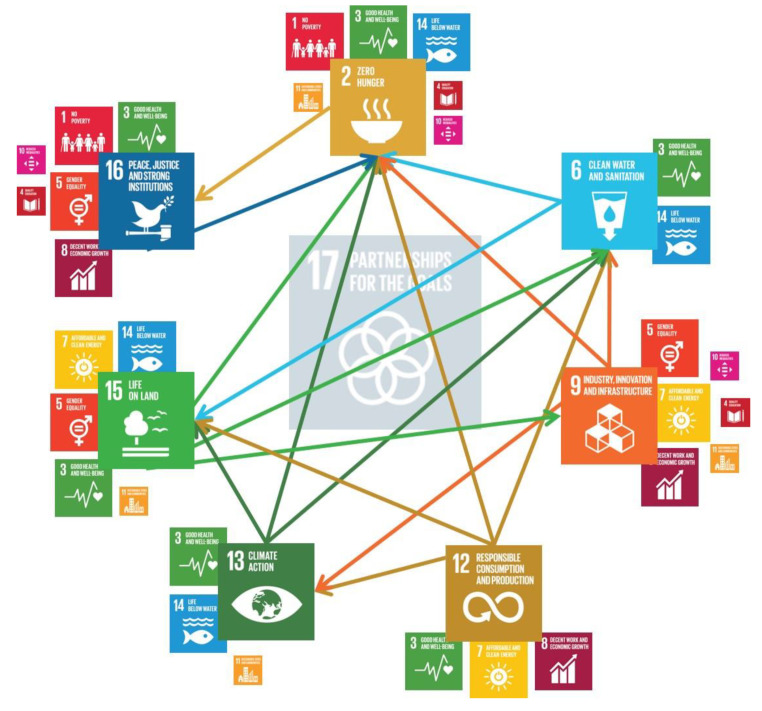
Links between sustainable development goals in the “edible insects’ idea”.

Insect farming, as a new production sector, could surely contribute to reaching several SDGs (Figure 2). 

The interaction map, while not exhaustive, can help in making effective policy in the insect sector within the paradigm of the SDGs and their respective targets. A new supply chain production could corroborate markets and increase incomes, as is could also be a spark in new market niches. Insect farming, as mentioned, does not need a large initial cost, both economically and in regard to expertise. Starting a proficient insect farm could be done with small initial costs, low price materials, and does not require high personnel expertise. Therefore, this practice could also be introduced in low-income countries with a minimum level of instruction and guide. The production of insects intended to be used as feed and food could surely reduce hunger and contribute to promoting peaceful and inclusive societies, and it could also contribute to reducing the economic gaps and increasing Gross Domestic Product (GDP) (SDG 1. End poverty in all its forms everywhere; SDG 10. Reduce inequality within and among countries). An improved society, which is less influenced by financial discrepancy, will reduce vulnerability and support equal rights. These can eventuate only in terms of monetary availability, but also in gender equality (SDG 5. Achieve gender equality and empower all women and girls) and level of education (SDG 4. Quality education). The inclusion of women is a milestone to be reached as soon as possible in several countries, and it will be even more impactful in rural communities. Insect farming, as a minilivestock, is not resource intensive, and it may be more accessible to women than other farming practices that are already culturally linked as male prerogatives. This has already been demonstrated in insect wild gathering, in which women and children play major roles, both as collectors and sellers. 

A healthier society with an increased economic capacity will lead to a decrease of food insecurity and susceptibility. A promotion of well-being and public health will be expected. New commercial capacity could contribute to increased incomes (SDG 8. Promote inclusive and sustainable economic growth, employment, and decent work for all), and the ability to access to better (nutritional) foods (SDG 3. Ensure healthy lives and promote well-being for all at all ages). In these topics, insect rearing could play a two-fold role, as an economic promoter (via new production chains) and nutritional food (compared to other animals’ products). Education and training will then follow as a necessary upgrade to reaching greater societal goals and community well-being (SDG 4. Quality education).

Insect’s farming could also contribute to a sustainable use of marine resources (SDG 14. Conserve and sustainably use the oceans, seas, and marine resources). Even though no aquatic insects are currently farmed, insects could contribute to the reduction of overfishing via the substitution (partially or totally) of fishmeal and fish oil in livestock feed. Indeed, a large portion of aquaculture is currently based on feed containing these two ingredients as proteins or fats, with an enormous environmental burden. 

On another environmental point of view, insect farming could indirectly contribute to increasing modern and sustainable energy services (SDG 7. Ensure access to affordable, reliable, sustainable, and modern energy) playing a role as an ingredient in bio-fuel production. In this term, insect farming will not contribute more in food–feed productions, but it may still contribute to reducing waste materials (and even do not compete with the feed–food supply chains as different—not allowed—substrates could be used in this process). The increase in alternatives energy resources could contribute to the reduced use of fossil fuels, contributing to reduced air pollution (SDG 11. Make cities inclusive, safe, resilient, and sustainable), and stopping several disagreements and controversies based on the economy of fuel.

## 4. Conclusions

Feeding the rapidly growing human population in sustainable ways is essential in increasing planetary health. Furthermore, these new productions (like insects) should also participate in reducing the negative environmental impacts already established and collaborated to shift to greener productions. In particular, edible insects have the potential to confer numerous benefits to people and the environment, in accordance with several SDGs. Close collaborations among all stakeholders, government, industry, and academia are required in order to succeed. Generating knowledge sharing networks, investing in interdisciplinary research, and developing sustainable policies will be necessary to capitalize on the benefits of edible insects in the future. Legislative frameworks and cultural barriers will also play their roles, along with market strategies, but we surely have to take advance of this opportunity to actively and indirectly contribute to so many SDGs to increase the global health status. At the same time, the SDG interdependencies can be translated into policy-making requirements, and can be useful in indicating a roadmap for how research can provide orientation for policy action.
